# Clinical and genetic features, surgical management, and longitudinal follow-up in two children with progressive osseous heteroplasia

**DOI:** 10.3389/fgene.2026.1883125

**Published:** 2026-10-02

**Authors:** Yuehan Yin, Binshan Zhao, Liying Sun, Wenyao Zhong, Dan Chen, Xiuli Zhao, Wen Tian

**Affiliations:** 1 Department of Hand Surgery, Clinical and Research Center for Congenital Hand Deformities and Rare Diseases, Beijing Jishuitan Hospital, Capital Medical University, Beijing Research Institute of Traumatology and Orthopaedics, Beijing, China; 2 Center for Rare Diseases, State Key Laboratory of Complex, Severe, and Rare Diseases, Peking Union Medical College Hospital, Chinese Academy of Medical Sciences, Beijing, China

**Keywords:** GNAS, POH, prognosis, progressive osseous heteroplasia, surgical treatment

## Abstract

**Background:**

Progressive osseous heteroplasia (POH) is an ultrarare disorder characterized by heterotopic ossification that progresses from the skin and subcutaneous tissues into deeper connective tissues. Most affected individuals harbor heterozygous inactivating variants in *GNAS*, whereas postzygotic mosaic variants have rarely been described. Evidence to guide surgical management remains limited.

**Methods:**

We retrospectively characterized two children with clinically diagnosed POH who underwent genetic testing and surgical treatment. Whole-exome sequencing was performed on peripheral blood and, when available, resected tissue, followed by PCR-Sanger sequencing and T-vector cloning. Case 1 underwent clinical and radiographic follow-up for 5 years; follow-up for Case 2 was limited to family-provided radiographs obtained at 2 years.

**Results:**

Case 1 harbored a possible postzygotic mosaic *GNAS* variant, NM_000516.7:c.-1_2del, with variant allele frequencies of 14.7% in peripheral blood and 40.56% in bone lesion tissue. The predominantly localized wrist lesion was macroscopically excised. A small radiographic recurrence appeared by the second postoperative year but progressed slowly thereafter; active wrist flexion was 5–10 ° at 5 years. Case 2 harbored NM_000516.7:c.565_568del (p. (Asp189MetfsTer14)), detected as a heterozygous variant in peripheral blood. The lesion diffusely involved neurovascular structures, tendon sheaths, the joint capsule, and adjacent bones, precluding complete excision. Family-provided radiographs at 2 years showed an extent of ossification broadly similar to that before surgery; no in-person clinical assessment was available.

**Conclusion:**

These cases provide additional clinical, molecular, and longitudinal information on POH and illustrate the importance of lesion distribution and resectability when interpreting surgical outcomes. The available postoperative findings cannot establish a causal relationship between variant status and prognosis because the patients differed in baseline disease extent, completeness of excision, follow-up duration, and outcome ascertainment. A possible association between mosaic burden and lesion distribution remains a hypothesis that requires evaluation in larger cohorts.

## 1 Introduction

Progressive osseous heteroplasia (POH) is an ultrarare genetic disorder characterized by heterotopic ossification that begins in the dermis or subcutaneous tissues and extends into deeper structures, including fascia, skeletal muscle, tendons, and ligaments (Kaplan et al., 1994a; b; Pignolo et al., 2015; Adegbite et al., 2008). Lesions usually appear during infancy or early childhood. Progression into deeper tissues may cause joint contracture, deformity, pain, skin breakdown, and severe functional impairment (Kaplan et al., 1994a; Pignolo et al., 2015).

Most individuals with POH harbor heterozygous inactivating variants in *GNAS*, a complex imprinted locus on chromosome 20q13.3 that encodes the alpha subunit of the stimulatory G protein (Gs α ) (Shore et al., 2002; Bastepe, 2018; Weinstein et al., 2001; Bastepe, 2007). Reduced Gs α signaling promotes osteogenic differentiation of mesenchymal progenitor cells and suppresses adipogenic differentiation (Lietman et al., 2005; Zhao and Ding, 2007). The phenotype associated with an inactivating *GNAS* variant is influenced by parental origin and tissue distribution. However, postzygotic mosaic variants in POH have rarely been reported.

No medical treatment reliably prevents or reverses POH. Surgery may be considered for circumscribed lesions that cause functional impairment, pain, skin breakdown, or compression of critical structures, but diffuse lesions are often incompletely resectable and may recur (Pignolo et al., 2015). Evidence to guide patient selection for surgery and the interpretation of long-term outcomes remains limited because POH is rare and published reports are heterogeneous.

We describe the clinical, molecular, surgical, and longitudinal findings of two children with POH. One child had a possible postzygotic mosaic *GNAS* variant with different variant allele frequencies across tissues, whereas the other had a heterozygous frameshift variant detected in peripheral blood. We use these cases to generate, rather than test, hypotheses about how tissue mosaicism, lesion distribution, and resectability may relate to clinical course.

## 2 Materials and methods

### 2.1 Study design, patients, and ethics

This retrospective case series included two children evaluated and treated at Beijing Jishuitan Hospital between 2020 and 2025. We reviewed clinical records, examination findings, radiographs, operative findings, histopathology, genetic test results, and postoperative follow-up data.

The study was reviewed and approved by the Beijing Jishuitan Hospital Ethics Committee under approval numbers K2024-365-00 and 201808-09 and was conducted in accordance with institutional requirements and the Declaration of Helsinki. Written informed consent for participation and for publication of clinical information and images was obtained from the patients’ parents or legal guardians.

Published clinical diagnostic criteria for POH were applied (Pignolo et al., 2015; Adegbite et al., 2008). The three major criteria were (1) superficial heterotopic ossification with progression into deep connective tissues, (2) no more than two features of Albright hereditary osteodystrophy other than heterotopic ossification, and (3) absence of parathyroid hormone resistance. Supporting findings included an inactivating *GNAS* variant, evidence of paternal inheritance, a reticular radiographic pattern of ossification, exclusive intramembranous ossification or combined intramembranous and endochondral ossification, lateralization in a dermomyotomal pattern, intrauterine growth restriction, leanness, and onset before 1 year of age (Pignolo et al., 2015). Available clinical records were reviewed for these features and for biochemical evidence of hormone resistance.

Outcomes of interest included lesion recurrence, radiographic progression, complications, reoperation, documented joint range of motion, and functional limitations. Range-of-motion values were abstracted only from contemporaneous clinical records and were not estimated from photographs. No validated patient-reported outcome measure or hand-function score was administered. Case 2 did not return for an in-person assessment at 2 years; only radiographs provided by the family were available.

### 2.2 Genetic analysis

Genomic DNA was extracted from fresh peripheral-blood samples from both probands and from fresh surgically resected bone-lesion tissue from Case 1 by proteinase K digestion followed by phenol-chloroform extraction. Only the peripheral-blood result is reported for Case 2. DNA concentration and purity were assessed using a NanoDrop 1000 spectrophotometer (Implen, Munich, Germany).

Whole-exome sequencing was performed by KingMed Diagnostics using an internally validated commercial workflow on an Illumina HiSeq 4000 platform. Paired-end 150-bp sequencing was performed. The service report indicated an approximate mean target-region depth of 1,000 × but did not provide per-specimen coverage metrics. Reads were aligned to the UCSC hg19 (GRCh37) human reference genome using BWA. Variants were called using the GATK software suite and annotated using the Ensembl Variant Effect Predictor. A minimum VAF threshold of 5% was applied for mosaic-variant screening. Variants with a minor allele frequency greater than 1% in gnomAD were excluded. ClinVar, dbSNP, HGMD, COSMIC, OMIM, and the published literature were used for clinical and functional annotation rather than as population-frequency databases. Catalogue numbers for the library-preparation and exome-capture kits, software versions, duplicate-marking details, target-coverage percentages, and contamination-check procedures were unavailable in the service-provider report and therefore could not be reported.

Variant allele frequency was defined as the proportion of high-quality variant-supporting reads among all high-quality reads at the variant locus. Reads were filtered at the reported thresholds of base quality ≥ 20 and mapping quality ≥ 30, and VAF values were exported from the KingMed bioinformatics pipeline. The underlying variant and reference read counts, total depth, strand distribution, and original software output were unavailable for independent review. The reported VAF values were not derived from T-vector clone counts.

Candidate *GNAS* variants were confirmed by PCR amplification and Sanger sequencing using transcript NM_000516.7 as the reference sequence. PCR was performed with LA Taq polymerase (Takara Bio, Kusatsu, Japan) in a 25- μ L reaction: 95 ° C for 3 min; 35 cycles of 94 ° C for 30 s, 60 ° C for 35 s, and 72 ° C for 50 s; and a final extension at 72 ° C for 8 min. PCR products were separated on 2% agarose gels. T-vector cloning and sequencing were used to distinguish the reference and deleted alleles in the available validation specimens. For Case 1, the validation primers were $5^{\prime }$ -ACC​GAC​ACC​CTC​CCC​TTC- $3^{\prime }$ (forward) and $5^{\prime }$ -GTC​TTA​CTG​TTC​CCG​AGG​CA- $3^{\prime }$ (reverse). For Case 2, the primers were $5^{\prime }$ -ACG​CAT​CTA​GAG​TTC​CCT​GA- $3^{\prime }$ (forward) and $5^{\prime }$ -TTG​GAA​AGA​GGG​CTC​AGA​GT- $3^{\prime }$ (reverse). Per-specimen clone counts and a study-specific ACMG/AMP assessment were not available and are not reported.

The parents of both probands were reported to have no clinical signs of heterotopic ossification. Parental biological samples were unavailable for genetic testing. Consequently, neither variant could be classified as inherited or *de novo*, and parental origin could not be assigned.

## 3 Results

### 3.1 Clinical findings

#### 3.1.1 Case 1

Case 1 was a girl aged 10 years and 4 months at presentation in August 2020. Her parents first noticed a solitary firm papule on the right heel when she was approximately 1 month old. Multiple painless papules, approximately the size of millet seeds, subsequently developed on the dorsal left wrist. Six years before presentation to our center, the wrist lesion was excised at another hospital and the defect was covered with a skin graft; histopathology showed mature trabecular bone. The lesion recurred approximately 1 year after that operation and gradually enlarged. Two years before presentation, it protruded through the skin and intermittently discharged purulent material. At presentation, the dorsal wrist lesion measured approximately 6 × 3 cm (Figure 1A). No family history of a similar disorder was reported.

**FIGURE 1:**
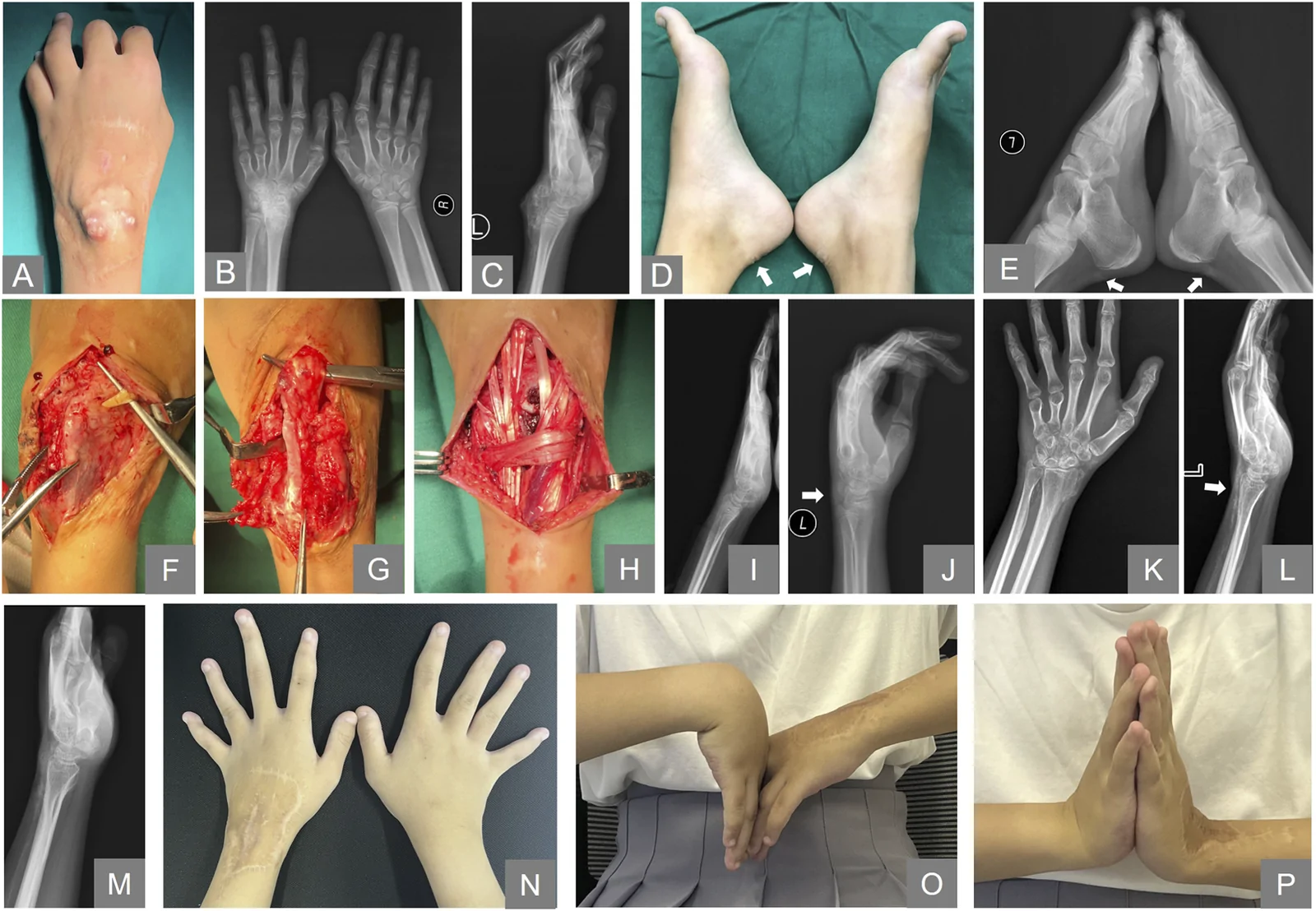
Clinical presentation, surgery, and follow-up of Case 1. **(A)** Preoperative dorsal view of the left wrist showing recurrent nodular lesions within the previous skin-graft area. **(B)** Bilateral hand radiograph showing shortened metacarpals. **(C)** Lateral radiograph of the left wrist showing dorsal heterotopic ossification. **(D)** Clinical appearance of both heels; arrows indicate palpable nodules near the Achilles tendon insertions. **(E)** Bilateral foot radiographs; arrows indicate subcutaneous ossification near the calcanei. **(F)** Intraoperative exposure showing heterotopic bone surrounding the extensor structures. **(G)** The elongated ossified lesion and involvement of the extensor retinaculum. **(H)** Operative field after lesion excision and reconstruction of the extensor retinaculum. **(I)** Radiograph 2 months after surgery showing no visible residual lesion. **(J)** Radiograph at postoperative year 2 showing a small recurrent radiopaque focus. **(K,L)** Anteroposterior and lateral radiographs at postoperative year four showing slight enlargement. **(M)** Lateral radiograph at postoperative year five showing little interval change. **(N)** Clinical appearance of the healed dorsal wrist at year 5. **(O)** Wrist flexion at year 5. **(P)** Wrist extension at year 5.

Examination showed multiple firm white nodules within the dorsal wrist skin-graft area, wrist stiffness, enlargement of the middle phalanx of the left index finger, and positive knuckle signs affecting the ring and little fingers. Preoperatively, active wrist extension was 60 ° , flexion was limited to the neutral position (0 ° ), and forearm pronation and supination were each 60 ° . Small nodules were present near both Achilles tendons (Figure 1D). Radiographs showed irregular heterotopic ossification on the dorsal left wrist, narrowing of some joint spaces, shortening of the fourth and fifth left metacarpals and the third to fifth right metacarpals, and subcutaneous ossification near both calcanei (Figures 1B–E).

Case 1 met the major ossification criterion and had supporting features including onset before 1 year of age, an inactivating *GNAS* variant, and intramembranous and/or endochondral bone formation. On 19 August 2020, serum calcium was 2.39 mmol/L (reference range, 2.25–2.67 mmol/L), serum phosphate was 1.57 mmol/L (reference range, 1.45–2.10 mmol/L), and alkaline phosphatase was 163 IU/L (reference range, 50–400 IU/L). Parathyroid hormone was not measured, and thyroid-function and other hormone-resistance test results were unavailable. Consequently, the absence of parathyroid hormone resistance could not be confirmed.

#### 3.1.2 Case 2

Case 2 was a boy aged 6 years and 5 months at presentation in July 2021. At approximately 1 year of age, multiple firm, painless nodules, approximately the size of millet seeds, appeared on the palmar aspect of the left hand over the third and fourth metacarpals. The lesions enlarged and extended along the ulnar side of the middle finger, causing progressive deformity and functional impairment. No family history of a similar disorder was reported.

The left middle finger was thickened and deviated radially, whereas the ring finger deviated ulnarly. Firm, irregular nodules measured approximately 2 × 1.5 cm over the third and fourth metacarpals and 4 × 0.5 cm along the ulnar side of the middle finger. Flexion and extension of the middle and ring fingers were restricted (Figure 2A). Radiographs showed irregular ossification between the third and fourth metacarpals, along the ulnar side of the middle finger, and around adjacent metacarpal and phalangeal bones (Figures 2B,C). Additional ossification was present in the soft tissues beneath the left calcaneus (Figure 2D). Together with the operative evidence of deep involvement described below, these findings supported the major criterion of superficial-to-deep progression. No clinical feature of AHO other than heterotopic ossification was identified, thereby fulfilling the second major criterion. The detected *GNAS* variant and histopathological evidence of bone and cartilage formation were supporting findings. On 30 July 2021, serum calcium was 2.44 mmol/L (reference range, 2.25–2.67 mmol/L), serum phosphate was 1.41 mmol/L (reference range, 1.45–2.10 mmol/L), and alkaline phosphatase was 189 IU/L (reference range, 50–400 IU/L). Parathyroid hormone was not measured, and thyroid-function and other hormone-resistance test results were unavailable. Thus, although hyperphosphatemia was absent, the absence of parathyroid hormone resistance could not be confirmed, and the third major criterion remained unassessed.

**FIGURE 2:**
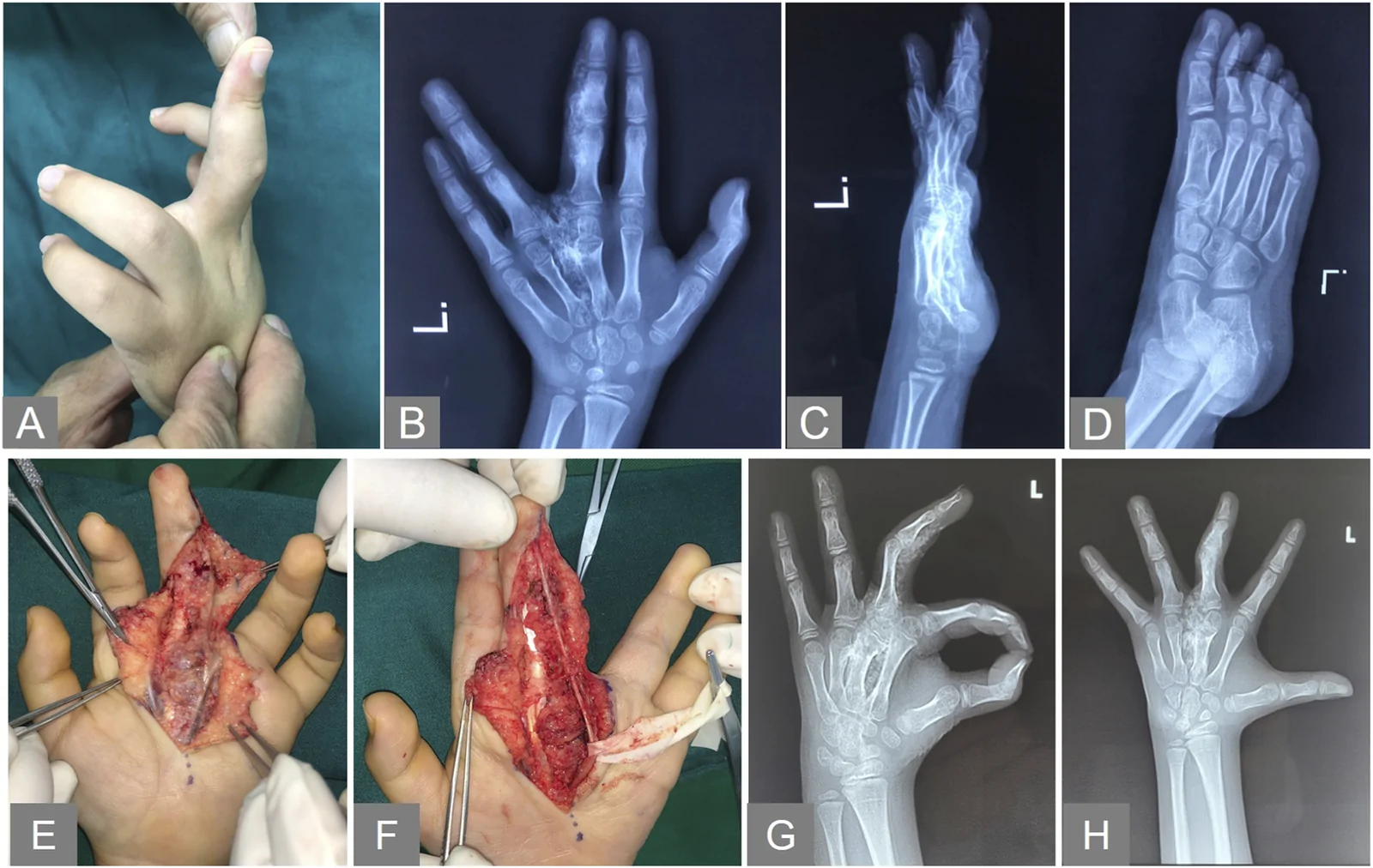
Clinical presentation, surgery, and follow-up of Case 2. **(A)** Preoperative appearance of the left hand showing middle-finger thickening and deformity. **(B,C)** Preoperative anteroposterior and lateral radiographs showing irregular ossification in the palmar hand and middle finger with involvement of adjacent bones. **(D)** Radiograph of the left foot showing ossification beneath the calcaneus. **(E,F)** Intraoperative views showing diffuse ossification around soft-tissue and neurovascular structures before and during excision. **(G,H)** Family-provided radiographs at postoperative year 2 showing an ossification extent broadly similar to that before surgery.

### 3.2 Molecular findings

Whole-exome sequencing identified *GNAS* (NM_000516.7):c.-1_2del in Case 1. The variant was present at a VAF of 14.7% in peripheral blood and 40.56% in bone lesion tissue, a distribution consistent with possible postzygotic mosaicism and relative enrichment in lesion tissue. PCR-Sanger sequencing and T-vector cloning confirmed the deleted and reference alleles (Figure 3A). The variant is catalogued in ClinVar (VCV002832188.4; rs2516794697) (National Center for Biotechnology Information, 2026) and is not presented here as novel.

**FIGURE 3:**
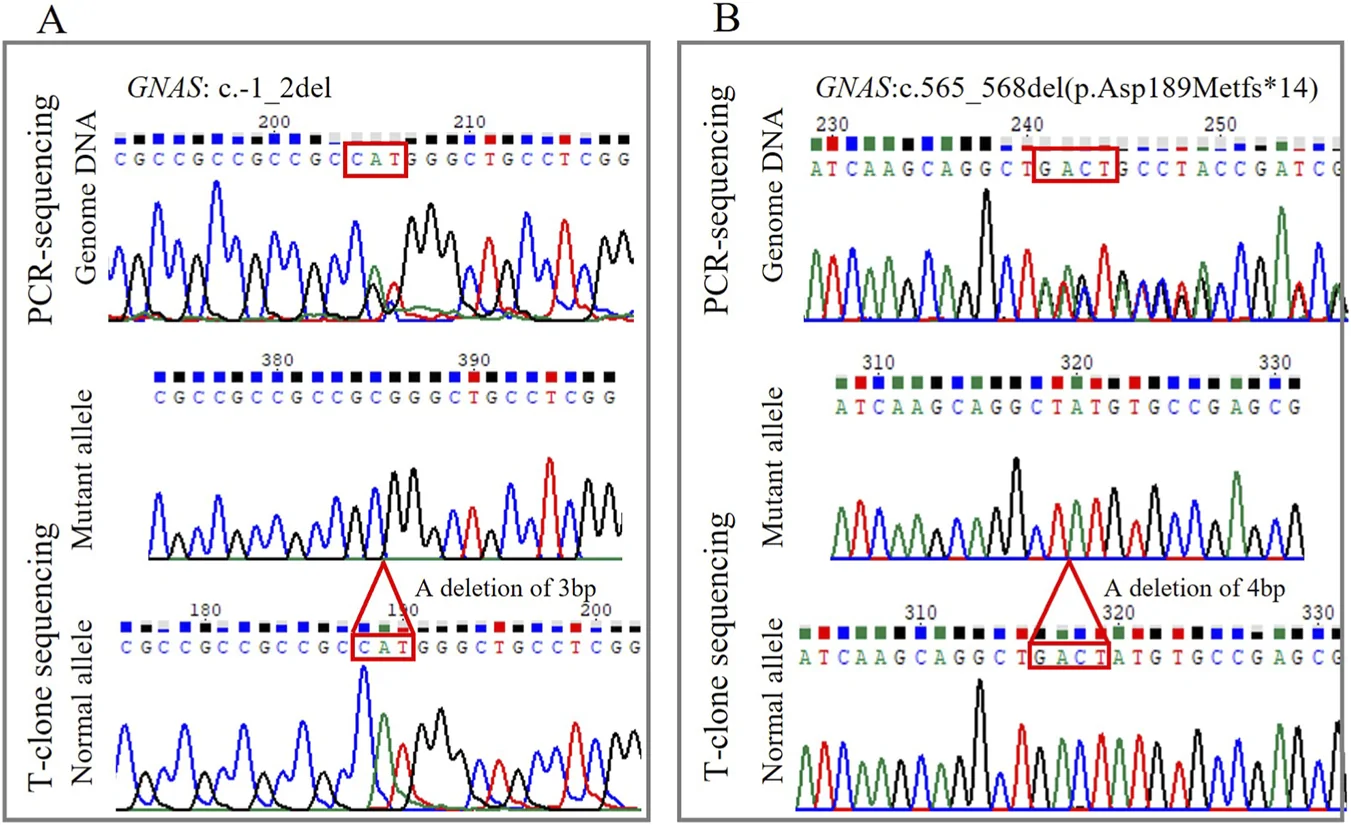
Molecular confirmation of the *GNAS* variants. **(A)** Case 1, showing NM_000516.7:c.-1_2del. The upper trace shows PCR-Sanger sequencing of genomic DNA; the middle and lower traces show T-vector clone sequencing of the mutant and reference alleles, respectively. The WES-reported VAFs were 14.7% in peripheral blood and 40.56% in bone lesion tissue; the specimen source represented by the upper genomic-DNA trace could not be assigned more specifically from the available records. **(B)** Case 2, showing NM_000516.7:c.565_568del (p. (Asp189MetfsTer14)). The upper trace shows PCR-Sanger sequencing of peripheral-blood genomic DNA; the middle and lower traces show T-vector clone sequencing of the corresponding mutant and reference alleles, respectively. Red boxes indicate the deleted reference nucleotides, and arrows indicate the deletion sites.

Whole-exome sequencing detected a heterozygous *GNAS* (NM_000516.7):c.565_568del (p. (Asp189MetfsTer14)) variant in peripheral blood from Case 2. PCR-Sanger sequencing and T-vector cloning confirmed the deleted and reference alleles (Figure 3B). This recurrent four-base-pair deletion has previously been reported in association with POH (Adegbite et al., 2008). Because parental samples were unavailable, the variant cannot be described as inherited, *de novo*, or of known parental origin in this patient.

The c.-1_2del variant overlaps the translation-initiation region and is predicted to disrupt normal translation initiation. Alternative translation initiation or altered transcript processing remains possible, but no RNA or protein assay was performed; the functional consequence therefore remains unconfirmed. The c.565_568del variant is predicted to introduce a frameshift and premature termination codon.

### 3.3 Surgical treatment and follow-up

In Case 1, surgery was undertaken because of progressive lesion enlargement, recurrent skin breakdown with intermittent discharge, wrist restriction, and functional and cosmetic concerns. The intermittent discharge did not by itself establish active infection at the time of surgery. The lesion formed an elongated, approximately 10-cm ossified structure around the middle-finger extensor tendon. It extended to the radial wrist capsule and encased the extensor retinaculum (Figures 1F,G). The macroscopically visible lesion was completely excised, and the extensor retinaculum was reconstructed (Figure 1H). Histopathology showed mature ectopic bone containing fatty and vascular tissue. A radiograph obtained 2 months after surgery showed no visible residual lesion (Figure 1I).

Annual radiographic follow-up showed a small recurrent radiopaque focus by the second postoperative year (Figure 1J). The focus enlarged slightly by year four and showed little radiographic change at year 5 (Figures 1K–M). The incision remained healed, with no protruding mass (Figure 1N). At 5 years, active wrist extension was 90 ° , active flexion was 5–10 ° , pronation was 80 ° , and supination was 90 ° (Figures 1O,P). No repeat operation or other postoperative complication was documented in the available follow-up record.

In Case 2, surgery was performed because of progressive deformity and restricted motion of the middle and ring fingers; quantitative preoperative finger range of motion was not recorded. Although the lesion was diffuse, its overall distribution between the third and fourth metacarpals and along the ulnar side of the middle finger suggested preoperatively that resection might be feasible. The patient and family expressed a strong preference to proceed with surgery. Intraoperatively, the ossification was found to diffusely surround the neurovascular bundle, tendon sheaths, joint capsule, and adjacent metacarpal and phalangeal bones (Figures 2E,F). Complete excision was therefore considered unsafe; accessible ossification around the neurovascular bundle, between the metacarpals, and along the ulnar side of the middle finger was removed. Histopathology showed cancellous bone, spindle-cell proliferation, nodular cartilage formation, and ossification without cellular atypia. The patient did not return for an in-person assessment at 2 years. Radiographs provided by the family showed an extent of ossification broadly similar to that on preoperative imaging (Figures 2G,H). Postoperative range of motion, symptoms, clinical deformity, complications, and reoperation status therefore could not be assessed.

Table 1 summarizes the clinical, molecular, surgical, and follow-up findings.

**TABLE 1:** Comparison of clinical, molecular, surgical, and follow-up findings.

Characteristic	Case 1	Case 2
Sex; age at presentation	Female; 10 years 4 months	Male; 6 years 5 months
Age at first lesion	Approximately 1 month	Approximately 1 year
Main distribution	Dorsal left wrist; bilateral heel/Achilles-region foci	Palmar left hand, middle finger, adjacent bones; left calcaneal focus
Baseline functional finding	Wrist extension 60 ° ; flexion limited to neutral (0 ° ); pronation/supination 60 ° /60 °	Restricted middle- and ring-finger motion; quantitative ROM not recorded
Relevant biochemical results	Calcium 2.39 mmol/L; phosphate 1.57 mmol/L; ALP 163 IU/L; PTH not measured	Calcium 2.44 mmol/L; phosphate 1.41 mmol/L (below reference range); ALP 189 IU/L; PTH not measured
*GNAS* variant (NM_000516.7)	c.-1_2del	c.565_568del (p. (Asp189MetfsTer14))
Variant distribution	Possible postzygotic mosaicism; blood VAF 14.7%, bone lesion VAF 40.56%	Heterozygous in peripheral blood; parental origin undetermined
Parental testing	Not performed; samples unavailable	Not performed; samples unavailable
Surgical indication	Progressive enlargement, recurrent skin breakdown with intermittent discharge, wrist restriction, and functional/cosmetic concerns	Progressive deformity and motion restriction; preoperative assessment suggested resection might be feasible; patient and family expressed a strong preference to proceed
Operative extent	Macroscopically complete excision and extensor-retinaculum reconstruction	Incomplete excision because of diffuse involvement of critical structures
Follow-up	5 years, clinical and radiographic	2 years, family-provided radiographs only
Recurrence/progression	Small radiographic recurrence by year 2; slow subsequent enlargement	Family-provided year-2 radiographs showed an extent broadly similar to that before surgery
Final documented function	At year 5: wrist extension 90 ° , active flexion 5–10 ° , pronation 80 ° , and supination 90 °	Not assessed in person at year 2
Complications/reoperation	No repeat operation or other complication described in the available follow-up record	Not assessable from family-provided radiographs alone

## 4 Discussion

This report describes two children with POH, including one with a possible postzygotic mosaic *GNAS* variant that overlaps the translation-initiation region. Published case reports continue to broaden the allelic spectrum associated with POH (Ma et al., 2023). The higher VAF in lesion tissue than in peripheral blood is consistent with postzygotic mosaicism and suggests relative enrichment of variant-bearing cells in the lesion. Whether this distribution contributes to tissue-level heterogeneity remains uncertain. Moreover, a comparison of two patients cannot establish that mosaic variants generally cause localized disease or predict better surgical outcomes.

The c.-1_2del variant is of particular interest because it overlaps the translation-initiation region of *GNAS*. Variants affecting the amino terminus can alter translation initiation or produce truncated Gs α proteins (Puzhko et al., 2011; Kozasa et al., 1988; Richard et al., 2013). Nevertheless, the molecular consequences considered here are predictive. Without RNA, protein, or cellular assays, this study cannot determine whether c.-1_2del causes loss of the canonical start codon, alternative initiation, altered transcript processing, or another functional effect. Accordingly, these possible mechanisms remain hypothetical rather than demonstrated.

The available postoperative findings differed, but several factors preclude attributing this difference to genotype. Case 1 had a predominantly localized wrist lesion that could be macroscopically excised, whereas Case 2 had diffuse disease involving neurovascular structures, tendon sheaths, the joint capsule, and adjacent bones, making complete excision unsafe. Case 1 underwent clinical and radiographic follow-up for 5 years, whereas the only 2-year follow-up information for Case 2 consisted of family-provided radiographs without an in-person clinical assessment. Baseline severity, anatomical distribution, completeness of excision, follow-up duration, and extent of outcome ascertainment are therefore major confounders. Notably, Case 1 also developed radiographic recurrence, although progression was slow after the second postoperative year.

Published experience suggests that well-circumscribed POH lesions may occasionally be removed with sustained clinical benefit, whereas diffuse lesions frequently recur or produce complications after surgery (Pignolo et al., 2015; Schrander et al., 2014). The present cases are consistent with that distinction but do not establish an indication for early radical excision. Surgical decisions should remain individualized and should consider pain, skin breakdown, functional restriction, neurovascular risk, lesion maturity and distribution, and the feasibility and morbidity of resection. Genetic findings may complement this assessment, but they should not independently determine the operative strategy.

These observations raise a preliminary, testable hypothesis that the tissue distribution and burden of *GNAS* variants may contribute to phenotypic heterogeneity in POH. They do not, however, support a dichotomous model in which postzygotic variants cause localized disease and favorable postoperative outcomes, whereas constitutional variants cause diffuse disease and poor outcomes. A previous report described extensive heterotopic ossification and limited surgical resectability in a patient with a germline *GNAS* variant (Schrander et al., 2014), but individual observations cannot establish an independent genotype–phenotype or genotype–prognosis association. Larger cohorts with systematic multi-tissue testing and standardized clinical assessment are needed to evaluate this hypothesis.

This study has several limitations. It includes only two clinically heterogeneous patients and has no control group or statistical analysis, resulting in a substantial risk that the observed differences occurred by chance. Follow-up duration and methods differed between the patients. No validated patient-reported outcome measure or hand-function score was administered, and quantitative preoperative finger motion was unavailable for Case 2. Moreover, Case 2 did not return for clinical examination at 2 years, limiting follow-up to family-provided radiographs and precluding assessment of postoperative function, symptoms, clinical deformity, complications, and reoperation. Parathyroid hormone was not measured in either patient, and thyroid-function and other hormone-resistance test results were unavailable, precluding a complete biochemical assessment of hormone resistance.

The molecular data also have important limitations. Parental samples were unavailable, preventing determination of inheritance and parental origin. Complete per-specimen WES coverage metrics, read-level counts and strand distribution, original VAF software output, and T-vector clone counts were unavailable, limiting independent verification and reproducibility of the molecular findings. No RNA, protein, or cellular functional assay of c.-1_2del was performed. Finally, any apparent genotype-associated effect cannot be separated from lesion distribution, completeness of excision, follow-up duration, and outcome ascertainment.

In conclusion, these cases add molecular and longitudinal clinical detail to the description of POH, particularly through the identification of a possible postzygotic mosaic *GNAS* c.-1_2del variant. They suggest the potential value of testing more than one tissue when mosaicism is suspected and illustrate the importance of considering lesion extent and resectability when interpreting surgical outcomes. Whether mosaic burden predicts lesion distribution or prognosis remains an open question that requires evaluation in larger, systematically phenotyped cohorts.

## Data Availability

The de-identified clinical, imaging, surgical, histopathological, and processed genetic data supporting the conclusions of this article will be made available by the corresponding authors upon reasonable request. Raw sequencing data and other potentially identifiable data from two children with an ultrarare disorder are not publicly available to protect participant privacy. Requests should be directed to Wen Tian (jsthand@163.com) and Xiuli Zhao (zhaoxiuli@pumch.cn) and will be considered subject to participant consent, institutional ethics requirements, privacy safeguards, and, where necessary, a data-use agreement.
